# Defining the relative performance of isothermal assays that can be used for rapid and sensitive detection of foot-and-mouth disease virus

**DOI:** 10.1016/j.jviromet.2017.08.013

**Published:** 2017-11

**Authors:** Emma L.A. Howson, Yohei Kurosaki, Jiro Yasuda, Masayoshi Takahashi, Hiroaki Goto, Ashley R. Gray, Valerie Mioulet, Donald P. King, Veronica L. Fowler

**Affiliations:** a The Pirbright Institute, Ash Road, Pirbright, Surrey, GU24 0NF, UK; b Institute of Biodiversity, Animal Health and Comparative Medicine, College of Medical, Veterinary & Life Sciences, Graham Kerr Building, University of Glasgow, G12 8QQ, UK; c Department of Emerging Infectious Diseases, Institute of Tropical Medicine, Nagasaki University, 1-12-4, Sakamoto, Nagasaki, 852-8523, Japan; d Toshiba Medical Systems Corporation, 1385, Shimoishigami, Otawara-shi, Tochigi, 324-8550, Japan

**Keywords:** Foot-and-Mouth disease, FMDV, Diagnostics, rRT-PCR, RT-LAMP, RT-RPA

## Abstract

This study describes the first multiway comparison of portable isothermal assays for the detection of foot-and-mouth disease virus (FMDV), benchmarked against real-time reverse transcription RT-PCR (rRT-PCR). The selected isothermal chemistries included reverse transcription loop-mediated isothermal amplification (RT-LAMP) and reverse transcription recombinase polymerase amplification (RT-RPA). The analytical sensitivity of RT-LAMP was comparable to rRT-PCR (10^1^ RNA copies), while RT-RPA was one log_10_ less sensitive (10^2^ RNA copies). Diagnostic performance was evaluated using a panel of 35 samples from FMDV-positive cattle and eight samples from cattle infected with other vesicular viruses. Assay concordance for RT-LAMP and RT-RPA was 86–98% and 67–77%, respectively, when compared to rRT-PCR, with discordant samples consistently having high rRT-PCR cycle threshold values (no false-positives were detected for any assay). In addition, a hierarchy of sample preparation methods, from robotic extraction to simple dilution of samples, for epithelial suspensions, serum and oesophageal-pharyngeal (OP) fluid were evaluated. Results obtained for RT-LAMP confirmed that FMDV RNA can be detected in the absence of RNA extraction. However, simple sample preparation methods were less encouraging for RT-RPA, with accurate results only obtained when using RNA extraction. Although the evaluation of assay performance is specific to the conditions tested in this study, the compatibility of RT-LAMP chemistry with multiple sample types, both in the presence and absence of nucleic acid extraction, provides advantages over alternative isothermal chemistries and alternative pen-side diagnostics such as antigen-detection lateral-flow devices. These characteristics of RT-LAMP enable the assay to be performed over a large diagnostic detection window, providing a realistic means to rapidly confirm positive FMD cases close to the point of sampling.

## Highlights

• Isothermal assays representative of RT-LAMP and RT-RPA were compared against rRT-PCR.
• Nine sample preparation methods were evaluated across all assays.
• RT-LAMP can detect FMDV in multiple sample types using simple preparation techniques.
• RT-LAMP can detect FMDV across a large diagnostic detection window.

## 1 Introduction

Molecular diagnostic assays such as PCR are now recognised as reliable detection methods for many viral pathogens, including foot-and-mouth disease (FMD) virus (FMDV: family *Picornaviridae*, genus *Aphthovirus*) (The World Organisation for Animal Health [OIE, (2012)], 2012). Endemic across many countries in Asia and Africa and parts of South America, FMD is highly contagious, with outbreaks in previously disease-free areas incurring severe economic damage (Knight-Jones and Rushton, 2013). Accurate and early diagnosis is therefore critical for the rapid enforcement of monitoring, control and eradication strategies. Laboratory-based real-time reverse transcription PCR (rRT-PCR) has become a widely accepted molecular tool for the detection and quantification of FMDV RNA, with diagnostic rRT-PCR assays developed for detection of all seven FMDV serotypes (A, O, C, Asia 1 and Southern African Territories [SAT] types 1–3) across a wide range of sample types (Callahan et al., 2002, Reid et al., 2002). Although relatively quick to perform, only taking a few hours to produce results (Shaw et al., 2007), sample transportation to laboratories is required which can be lengthy and consequently delay critical decision making.

In order to address this gap, research initiatives have prioritised the transition of molecular assays into formats suitable for deployment closer to suspect cases of FMD. For instance, rRT-PCR has been transferred onto a portable platform which integrates RNA extraction, thermal cycling and result reporting (Madi et al., 2012), and trialled successfully in field settings (Howson et al., 2015). However, in order to maintain precise thermal regulation for PCR, expensive instrumentation is necessary. As an alternative, a number of isothermal chemistries have been adapted for detection of FMDV. To date, there are 17 FMDV-specific publications detailing four isothermal chemistries: reverse transcription loop-mediated isothermal amplification ([RT-LAMP] Dukes et al., 2006, Li et al., 2009, Ranjan et al., 2014, Shao et al., 2010, Chen et al., 2011a, Chen et al., 2011b, Yamazaki et al., 2013, Madhanmohan et al., 2013, Guan et al., 2013, Waters et al., 2014, Ding et al., 2014, Howson et al., 2015), reverse transcription recombinase polymerase amplification ([RT-RPA] Abd El Wahed et al., 2013), nucleic acid sequence based amplification ([NASBA] Collins et al., 2002, Lau et al., 2006, Lau et al., 2007) and reverse transcription helicase dependent amplification ([RT-HDA] Jingwei et al., 2014, Jing et al., 2013). Two of these (RT-LAMP and RT-RPA) have been transitioned into portable formats and successfully trialled in endemic settings (Abd El Wahed et al., 2013, Howson et al., 2015).

Understanding the diagnostic performance of new and improved assay formats is usually undertaken by pairwise concordance testing against an established reference test. Whilst this approach is useful for benchmarking assays in isolation, it does not always permit an understanding of how assays sit in the context of other similar tests. For instance, although rRT-PCR is routinely chosen as the reference test, the use of different rRT-PCR reagents, conditions and rRT-PCR cut-off values results in variations between laboratory validation studies.

Furthermore, although these isothermal assays are regularly reported as suitable for point-of-care testing (POCT) the majority of FMDV-specific isothermal assays have been validated in laboratory settings, using nucleic acid extracted using either robots or manual extraction kits not suitable for field use. A number of alternative simple sample preparation methods have been developed: preparation of serum, epithelial suspensions and oesophageal-pharyngeal (OP) fluid by dilution in nuclease-free water (NFW) has shown to produce accurate RT-LAMP results (Waters et al., 2014, Howson et al., 2015) and simple elution from disposable immuno-chromatographic strip tests has been successful for sample preparation for rRT-PCR (Fowler et al., 2014). However, these methods are yet to be compared.

In this article we present the first comparison of different isothermal assay formats for detection of FMDV, using a panel of samples benchmarked against a recommended molecular assay. Assays included representatives of RT-LAMP and RT-RPA, in addition to a selection of sample preparation methods. These tests and sample preparation methods were selected based on existing literature (Abd El Wahed et al., 2013, Howson et al., 2015) and because they represent the most realistic options for FMDV-specific POCT deployment.

## 2 Materials and methods

### 2.1 Ethics statement

All clinical samples utilised in this study were either archival samples from previous experimental studies approved by The Pirbright Institute Ethical Review Committee under the Animal Scientific Procedures Act (ASPA) 1986 (as amended), or comprised samples submitted by endemic countries to The Food and Agriculture Organization of the United Nations (FAO) World Reference Laboratory for FMD (WRLFMD) at The Pirbright Institute (TPI), UK.

### 2.2 Reference real-time RT-PCR (rRT-PCR)

The one-step rRT-PCR, used as the reference test, employed primers and probes as previously described to target the highly conserved 3D^pol^-coding region of the FMDV genome (Callahan et al., 2002). Reagents, parameters and thermal cycling were as reported in Shaw et al. (2007). rRT-PCR reactions were performed in duplicate on a bench top real-time PCR machine (Stratagene Mx3005P™: Agilent Technologies, CA, USA) (Table 1).

**Table 1:** Molecular assay formats compared.

Assay	Method	Reagents	Reagent form	Supplier	Primers and Probes	Platform
Laboratory-based rRT-PCR	rRT-PCR	Shaw et al., 2007	Wet	Invitrogen (CA, USA)	Callahan et al., 2002	Mx3005P™
rRT-LAMP-d-wet	rRT-LAMP	ISO–001 + AMV	Wet	OptiGene Ltd.	Dukes et al., 2006	Genie^®^ II
rRT-LAMP-d-dry	rRT-LAMP	ISO-001 (*AMV included)	Lyophilised	OptiGene Ltd.	Dukes et al., 2006^a^	Genie^®^ II
RT-LAMP-LFD-d-wet	rRT-LAMP-LFD	ISO–001 + AMV	Wet	OptiGene Ltd.	Dukes et al., 2006	Genie^®^ II
RT-LAMP-LFD- D-dry	rRT-LAMP-LFD	ISO-001 (*AMV included)	Lyophilised	OptiGene Ltd.	Dukes et al., 2006^a^	Genie^®^ II
rRT-LAMP-T-wet	rRT-LAMP	ISO–001 + AMV	Wet	OptiGene Ltd.	Based on Shao et al., 2010	Genie^®^ II
rRT-RPA-exoRT	rRT-RPA	TwistAmp^®^ exo RT	Lyophilised	TwistDx Ltd.	Abd El Wahed et al., 2013	Genie^®^ II
rRT-RPA-nfo	rRT-RPA	TwistAmp^®^ nfo + RT	Lyophilised	TwistDx Ltd.	Abd El Wahed et al., 2013	Genie^®^ II

a Primers, probes and Avian Myeloblastosis Virus (AMV) were lyophilised within LAMP reagents. (rRT): real-time reverse transcription; (LAMP): loop-mediated isothermal amplification using either (D) Dukes et al. (2006) or (T) Toshiba Medical Systems Corporation primers; (LFD): molecular lateral-flow device; (RPA) recombinase polymerase amplification using either (exoRT) TwistAmp^®^ exo RT or (nfo) TwistAmp^®^ nfo reagents; (RT) reverse transcriptase.

### 2.3 RT-LAMP

#### 2.3.1 Real-time RT-LAMP (rRT-LAMP)

rRT-LAMP was performed using either Dukes et al. (2006) primers in both wet (rRT-LAMP-d-wet) and lyophilised (rRT-LAMP-d-dry) formats as previously described (Howson et al., 2015), or using primers from Toshiba Medical Systems Corporation [TMSC] (rRT-LAMP-T-wet). rRT-LAMP-T-wet was performed in a total reaction mixture of 25 μl, containing: 15 μl isothermal mastermix ISO-001 (OptiGene Ltd, Horsham, UK.), 2.5 μl TMSC FMDV primer mix (a proprietary RT-LAMP primer mix targeting the 3D^pol^-coding region), 0.15 U Avian Myeloblastosis Virus (AMV) reverse transcriptase (RT) (OptiGene Ltd.), 5 μl sample and made up to total volume with NFW. For all rRT-LAMP formats, reactions were performed in duplicate as previously described (Howson et al., 2015) using a Genie^®^ II (OptiGene Ltd.), with rRT-LAMP-T-wet incubated at 63 °C for 30 min. Time to positivity (T_P_) and anneal temperature (T_a_) calculations were automated using Genie^®^ Explorer v0.2.1.1 software (OptiGene Ltd.). Samples were called positive if amplification had occurred and the rRT-LAMP product annealed in the amplicon-specific temperature range (87.5–89.5 °C for Dukes et al. (2006) primers (Howson et al., 2015), 87.7-89.7 °C for TMSC primers)(Table 1).

#### 2.3.2 RT-LAMP-D combined with lateral-flow detection (RT-LAMP-d-LFD)

RT-LAMP-LFD was performed using both wet (*RT-LAMP-d-LFD-wet*) and lyophilised (*RT-LAMP-d-LFD-dry*) formats as previously described using modified Dukes et al. (2006) LAMP primers (Waters et al., 2014, Howson et al., 2015). Reactions were performed in duplicate on a Genie^®^ II and results were visualised using PCRD-2 lateral-flow devices (Abingdon Health, York, UK) as per manufacturer’s instructions and as previously described (Waters et al., 2014, Howson et al., 2015). For all images shown, the upper band represents the LFD control line (C), and lower band the test line (T) in respect to the loading pad at the bottom (Table 1).

### 2.4 Real-time RT-RPA (rRT-RPA)

#### 2.4.1 TwistAmp^®^ exo RT kit (rRT-RPA-exoRT)

rRT-RPA was performed using the TwistAmp^®^ exo RT kit (TwistDx Ltd., Cambridge, UK), with primers and probes as previously published (Abd El Wahed et al., 2013). Reactions were performed in duplicate at 42 °C for 20 min using a Genie^®^ II, with inversion at 5 min to mix. T_P_ was defined when reactions reached a threshold increase of δR 1500 (Table 1).

#### 2.4.2 TwistAmp^®^ nfo (rRT-RPA-nfo)

the TwistAmp^®^ nfo kit (TwistDx Ltd.) was used as manufacturer’s instructions, with the addition of 10 U RT Transcriptor (Roche, Mannheim, Germany). Primers and probes were as previously published (Abd El Wahed et al., 2013). Reactions were performed in duplicate at 39 °C for 40 min, with inversion at 4 min to mix. T_P_ was calculated as above (Table 1).

### 2.5 Comparison in the relative performance of isothermal assays

The analytical sensitivity of reactions was determined using an artificial RNA standard. This was produced as previously described (Howson et al., 2015) from *in vitro* transcription using a RT-PCR template generated from FMDV cell culture isolate O/UKG/35/2001. The standard (10^6^–10^0^ copies) was prepared in 0.1 μg/ml carrier RNA.

To determine the relative diagnostic performance of the different assays, RNA was extracted from a panel of clinical FMDV samples (Table 2) that had previously been submitted to WRLFMD. These samples comprised 35 FMDV-positive samples, representing five serotypes (O, A, SAT 1, SAT 2 and Asia 1) from ten countries and eight epithelial suspension samples representing viruses that cause similar characteristic lesions to FMDV: swine vesicular disease virus (SVD: UKG/24/1972; UKG/50/1972; UKG/51/1972; UKG/68/1972) and two serotypes of vesicular stomatitis virus (VSV: Indiana 1 [VSIV] n = 2; New Jersey [VSNJV] n = 2). RNA was extracted using a MagMAX™-96 Viral RNA Isolation Kit (Thermo Fisher Scientific, MA, USA) following an automated procedure on a KingFisher™ Flex (Thermo Fisher Scientific). RNA was extracted from 50 μl sample and eluted in a final volume of 90 μl MagMAX™-96 Viral RNA Isolation Kit elution buffer (Thermo Fisher Scientific).

**Table 2:** FMDV clinical samples used for diagnostic comparison of isothermal assays.

Virus	Serotype	Sample	Topotype	Lineage	Location	Sample Type
FMDV	O	HKN/12/2015	CATHAY	unnamed	Hong Kong	ES
		IRN/26/2015	ME-SA	PanAsia-2^BAL−09^	Iran	ES
		KUW/1/2016	ME-SA	PanAsia-2^BAL−09^	Kuwait	ES
		KUW/4/2016	ME-SA	PanAsia-2^BAL−09^	Kuwait	ES
		PAK/30/2015	ME-SA	PanAsia-2^BAL−09^	Pakistan	ES
		PAK/32/2015	ME-SA	PanAsia-2^BAL−09^	Pakistan	ES
		PAK/34/2015	ME-SA	PanAsia-2^BAL−09^	Pakistan	ES
		PAT/4/2015	ME-SA	PanAsia	Palestine	ES
	A	IRN/21/2015	ASIA	G-VII	Iran	ES
		IRN/24/2015	ASIA	Iran-05^SIS−10^	Iran	ES
		SAU/8/2015	ASIA	G-VII	Saudi Arabia	ES
		PAK/31/2015	ASIA	Iran-05^FAR−11^	Pakistan	ES
		PAK/56/2015	ASIA	Iran-05^FAR−11^	Pakistan	ES
		TAN/15/2013	AFRICA	G-I	Tanzania	ES
		TAN/71/2012	AFRICA	G-I	Tanzania	ES
	SAT 1	TAN/22/2014	I (NWZ)	unnamed	Tanzania	ES
		TAN/29/2013	I (NWZ)	–	Tanzania	ES
		TAN/23/2013	I (NWZ)	–	Tanzania	ES
		TAN/50/2012	I (NWZ)	unnamed	Tanzania	ES
		KEN/26/2008	I (NWZ)	–	Kenya	ES
		KEN/9/2009	I (NWZ)	–	Kenya	ES
		KEN/12/2009	I (NWZ)	–	Kenya	ES
	SAT 2	ZIM/9/2015	II	unnamed	Zimbabwe	ES
		ZIM/21/2015	II	unnamed	Zimbabwe	ES
		TAN/3/2011	IV	IV	Tanzania	ES
		TAN/64/2012	IV	unnamed	Tanzania	ES
		TAN/14/2012	IV	unnamed	Tanzania	ES
		TAN/19/2012	IV	unnamed	Tanzania	ES
		KEN/2/2007	IV	–	Kenya	ES
		SUD/7/2014	VII	Alx-12	Sudan	ES
	Asia 1	IRN/20/2015	ASIA	Sindh-08	Iran	ES
		PAK/33/2015	ASIA	Sindh-08	Pakistan	ES
		PAK/28/2015	ASIA	Sindh-08	Pakistan	OF
		PAK/29/2015	ASIA	Sindh-08	Pakistan	OF
		PAK/43/2015	ASIA	Sindh-08	Pakistan	ES

(FMDV): foot-and-mouth disease virus; ES (epithelial suspension); OF (oral fluid).

### 2.6 Comparison of sample preparation methodologies

To compare sample preparation methods, three decimal dilution series (10^−1^ to 10^−8^) of FMDV (cell culture isolate O/UAE/2/2003) were prepared in bovine epithelial suspensions (cattle tongues collected from a UK abattoir were prepared at 10% [w/v] in M25 phosphate buffer: 35 mM Na_2_HPO_4_ , 5.7 mM KH_2_PO_4_, pH 7.6), bovine serum (obtained from a UK abattoir) and bovine OP fluid (archival experimental samples from TPI). Aliquots were stored at −80 °C until use. All sample preparation methods were performed on rRT-LAMP-d-wet and rRT-RPA-exoRT, adding 5 μl of template sample to each assay.

A hierarchy of sample preparation methods were selected to compare, ranging from sophisticated laboratory nucleic extraction methods to simpler methods such as dilution of samples in NFW. Three nucleic extraction methods were tested. The MagMAX™-96 Viral RNA Isolation Kit was used as the reference sample procedure (MagMAX™-96 Viral RNA Isolation Kit/KingFisher™ Flex system as described above). The kit was also evaluated using a manual protocol, following manufacturer’s manual guidelines with a DynaMag™- Spin magnet (Thermo Fisher Scientific). The third extraction method evaluated was the QIAamp^®^ Viral RNA Mini Kit (Qiagen, Hilden, Germany), which was used according to manufacturer’s guidelines. RNA was extracted from 140 μl of sample (using the spin column protocol) and eluted in a final volume of 60 μl in QIAamp^®^ buffer AVE (Qiagen).

In addition, a number of simple sample preparation methods were selected as they had been previously described as simple techniques for PCR inhibitor removal (Walsh et al., 1991, Ochert et al., 1994, Wu and Kado, 2004, Fowler et al., 2014, Waters et al., 2014, Howson et al., 2015). The simplest of techniques tested was the dilution of samples 1 in 5, 1 in 10 or 1 in 20 in NFW. Secondly, syringe filters were evaluated by diluting samples 1 in 5 in NFW and passing 1 ml of sample through a Acrodisc^®^ 25 mm syringe filter (w/0.1 um Supor^®^ Membrane) (Pall Life Sciences, MI, USA). Thirdly, Chelex^®^ 100 was evaluated by adding 50 μl of 50% (w/v) Chelex^®^ 100–500 μl of diluted sample (1 in 5 in NFW). Samples were vortexed, allowed to settle and the supernatant used in assays. For Chelex^®^ 100 (heat), samples were heated at 56 °C for 10 min prior to processing as previously stated. Finally, extraction from antigen-detection lateral-flow devices (Ag-LFDs) was evaluated (epithelial material only), by adding 200 μl of each suspension to SVANODIP^®^ FMDV-Ag-LFDs (Ferris et al., 2009; Boehringer Ingelheim, Bracknell, UK) and incubating at room temperature (25 °C) for 72 h. Nucleic acid was extracted from the loading pad and wicking strip of the Ag-LFDs as previously described (Fowler et al., 2014).

## 3 Results

### 3.1 Comparison in the performance of isothermal assays

**Fig. 1:**
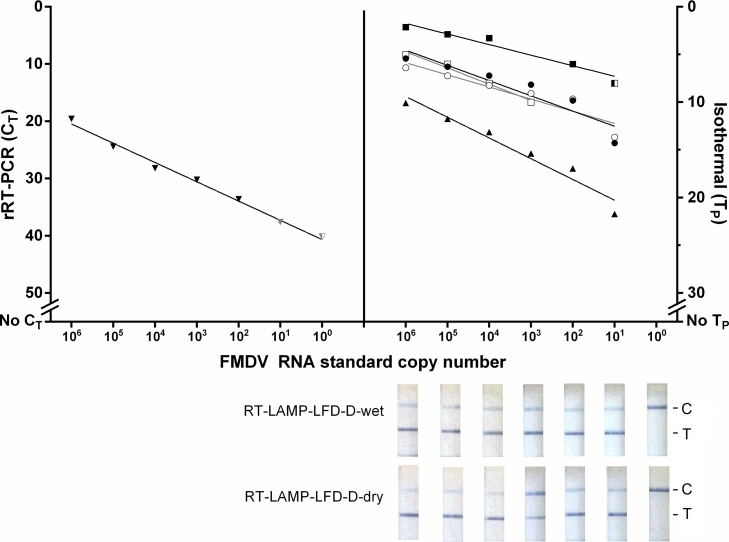
Analytical sensitivity of seven isothermal assay formats using a RNA standard. Results were compared to the diagnostic laboratory-based real-time reverse transcription (rRT)-PCR (▼). ● rRT-loop-mediated isothermal amplification using Dukes et al. (2006) primers (rRT-LAMP-D)-wet; ○rRT-LAMP-d-dry; ■ rRT-recombinase polymerase amplification (RPA)-exoRT;  □rRT-RPA-nfo; ▲ rRT-LAMP using Toshiba primers (rRT-LAMP-T)-wet. Half shaded points represent samples where one duplicate was positive and the other negative; grey shaded points (for rRT-PCR) represent C_T_ values that were over the diagnostic cut-off value of C_T <_32 (Shaw et al., 2007). For the molecular lateral-flow devices (LFD), the presence of a test (T) and control (C) line signifies a positive result; the presence of a single control band signifies a negative result (C). For all assays, both duplicates had to be positive for the sample to be regarded as a positive.

The analytical sensitivities of rRT-PCR, RT-LAMP (all formats) and rRT-RPA-exoRT, using the RNA standard, were 10^1^, 10^1^, and 10^2^ copies/μl, respectively (Fig. 1). Two log_10_ reductions in analytical sensitivity was evident for rRT-RPA-nfo compared to rRT-PCR, detecting down to 10^3^ copies/μl (Fig. 1).

**Fig. 2:**
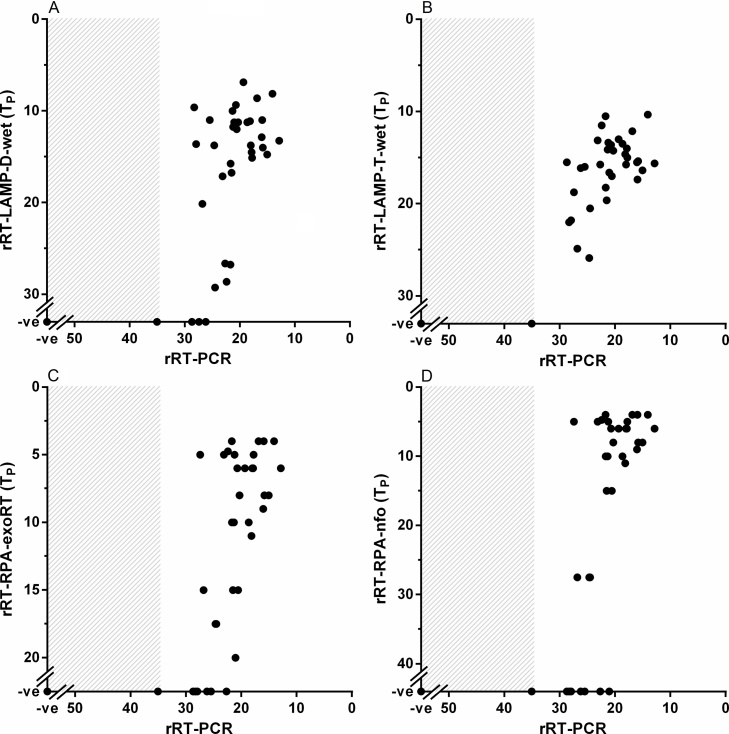
Concordance between the diagnostic laboratory-based real-time reverse transcription (rRT)-PCR and isothermal assays using 43 clinical samples: rRT-loop-mediated isothermal amplification using Dukes et al. (2006) primes (rRT-LAMP-D)-wet (A), rRT-LAMP using Toshiba primers (rRT-LAMP-T)-wet (B), rRT-recombinase polymerase amplification (RPA)-exoRT (C) and rRT-RPA-nfo (D). Grey box highlights rRT-PCR C_T_ values over the diagnostic threshold of C_T_ < 32 (Shaw et al., 2007). Lyophilisation of rRT-LAMP-D did not impact assay performance.

For diagnostic performance, the concordance between each isothermal assay and rRT-PCR for clinical samples (n = 43) were as follows: rRT-LAMP-d-wet (38/43 [88%]), rRT-LAMP-d-dry (38/43 [88%]), RT-LAMP-LFD-d-wet (37/43 [86%]), RT-LAMP-LFD-d-dry (37/43 [86%]), rRT-LAMP-T-wet (42/43 [98%]), rRT-RPA-exoRT (33/43 [77%]) and rRT-RPA-nfo (29/43 [67%]); false negative samples consistently had high rRT-PCR C_T_ values (Fig. 2) with no false positives detected for any test. All assays yielded negative results against SVDV and VSV isolates (data not shown).

### 3.2 Comparison of sample preparation methodologies

**Fig. 3:**
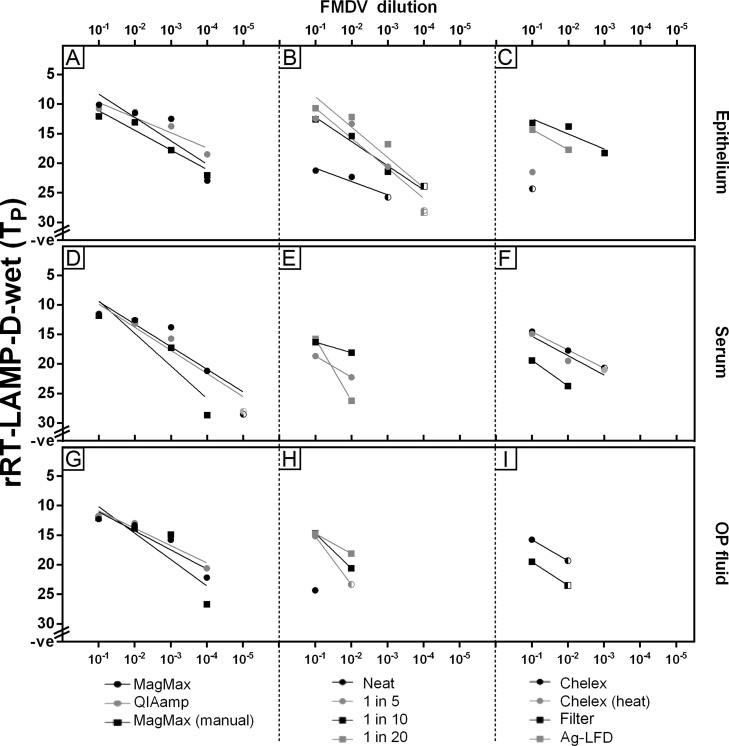
Comparison of sample preparation methods for real-time reverse transcription loop-mediated isothermal amplification using Dukes et al. (2006) primers (rRT-LAMP-d-wet). The top row (A-C) presents results for epithelial suspensions; the middle for serum samples (D-F); the bottom for oesophageal-pharyngeal (OP) fluid (G-I). A total of 11 sample preparation methods were trialled: (A,D,G): RNA extraction methods; (B,E,H): use of neat samples or dilution in nuclease-free water; (C,F,I): 1 in 5 dilutions subjected to further treatment using Chelex^®^ or syringe filtering. (Extraction from antigen-detection lateral-flow devices [Ag-LFD] was trialled for neat epithelial suspensions only) Half shaded points represent samples where one duplicate was positive and the other negative; both duplicates had to be positive for the sample to be regarded as a positive.

**Fig. 4:**
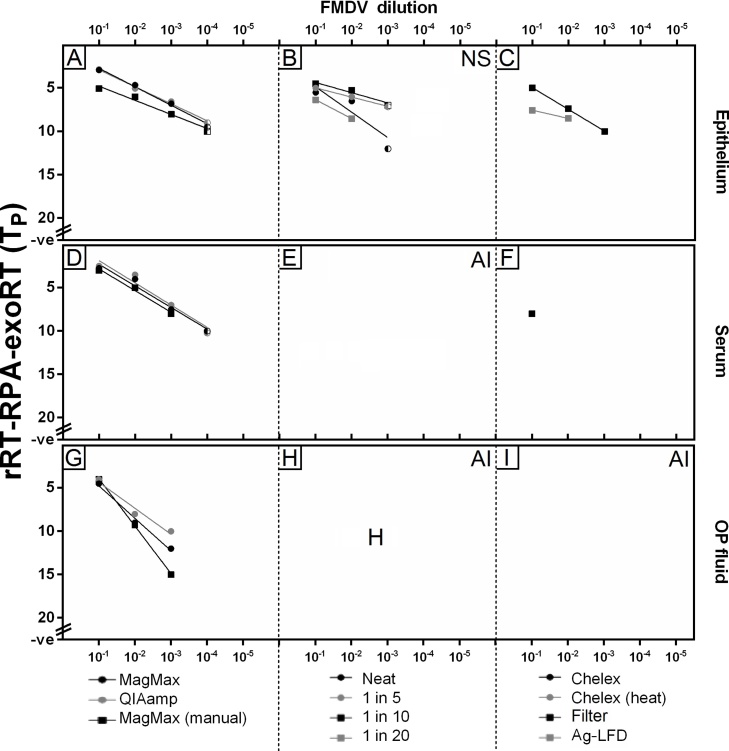
Comparison of sample preparation methods for real-time reverse transcription recombinase polymerase amplification using TwistAmp^®^ exo RT kit (rRT-RPA-exoRT). Sample preparation methods were trialled across three sample types: epithelial suspensions (A-C), serum samples (D-F) and oesophageal-pharyngeal (OP) fluid (G-I). Eleven sample preparation methods were tested: (A,D,G): RNA extraction methods; (B,E,H): use of neat samples or dilution in nuclease-free water; (C,F,I): 1 in 5 dilutions subjected to Chelex^®^ or syringe filtering (antigen-detection lateral-flow devices [Ag-LFD] extraction was trialled for neat epithelial suspensions only). NS: non-specific amplification detected (signified by amplification present in known negative samples); AI: assay inhibition. Half shaded points represent samples where one duplicate was positive and the other negative; both duplicates had to be positive for the sample to be regarded as a positive.

Nine simple methods were compared for preparation of samples prior to molecular analysis, using the MagMAX™-96 Viral RNA Isolation Kit/KingFisher™ Flex system as the reference sample preparation procedure. For both rRT-LAMP-d-wet and rRT-RPA-exoRT, the use of simple extraction kits (QIAamp^®^ and MagMAX™-96 Viral RNA Isolation Kit [manual]) achieved comparable analytical sensitivity across all sample types to the reference sample preparation procedure (Figs. 3A [epithelium], 3D [serum], 3G [OP fluid] and 4A [epithelium], 4D [serum], 4G [OP fluid]). For rRT-LAMP-d-wet, the direct use of epithelial samples (1 in 5, 1 in 10 or 1 in 20 dilution in NFW) resulted in a one log_10_ reduction in limit of detection (LOD) compared to the use of extraction kits (Fig. 3B). When epithelial samples were eluted from Ag-LFDs (which were positive up to 10^−3^ of the dilution series) or subjected to either Chelex^®^ 100 treatment or syringe filtering (after 1 in 5 dilution in NFW), a further decrease in LOD was observed (Fig. 3C). A one log_10_ reduction was also evident for rRT-RPA-exoRT when using epithelial samples directly (using dilution in NFW) (Fig. 4B) compared to extracted RNA (Fig. 4A), however non-specific amplification was evident in negative samples; this was removed by pre-processing of 1 in 5 dilutions through a syringe filter (Fig. 4C).

For serum, a two log_10_ reduction in LOD was evident when serum was diluted prior to analysis in rRT-LAMP-d-wet (Fig. 3E) compared to the use of extracted RNA (Fig. 3D). When serum was added neat to reactions rRT-LAMP-d-wet was inhibited (Fig. 3E). Following 1 in 5 dilution of serum in NFW, the use of Chelex^®^ 100 (heat) improved LOD, with only a one log_10_ reduction in LOD evident compared to the use of extracted RNA (Fig. 3F). For rRT-RPA-exoRT, the use of simple sample preparation techniques for serum resulted in assay inhibition (Fig. 4E and 4F), with extraction required (using any of the three RNA extraction methods tested) in order for amplification to be observed (Fig. 4D).

For OP fluid, again a two log_10_ reduction in LOD for rRT-LAMP-d-wet was evident when OP fluid was diluted in NFW (1 in 10 or 1 in 20) prior to analysis (Fig. 3H), compared to the use of extracted RNA (Fig. 3G). Neat OP fluid and OP fluid samples diluted 1 in 5 (with or without Chelex^®^ 100 treatment or syringe filtering), reduced the analytical sensitivity of rRT-LAMP-d-wet (Fig. 3H and 3I), with a three log_10_ reduction in LOD evident compared to the use of extracted RNA. For rRT-RPA-exoRT, extraction of RNA from OP fluid was required (Fig. 4G), with the use of simple sample preparation methods (either neat, using dilutions [with or without Chelex^®^ 100 or syringe filter treatment]) resulting in assay inhibition (Fig. 4H and I).

## 4 Discussion

The requirement for rapid and accurate diagnosis of FMD has led to an increased interest in evaluating simple-to-use diagnostic platforms that can be deployed closer to suspect cases of FMD. For instance, field-ready rRT-PCR, rRT-LAMP, RT-LAMP-LFD and rRT-RPA have been successfully trialled in endemic settings, allowing for accurate detection of FMDV *in situ* (Abd El Wahed et al., 2013, Howson et al., 2015). However, based on the lack of formal comparisons between these different assays, in addition to the lack of standardisation in assay evaluation, it is difficult to accurately gauge the best isothermal test based on independent publications at present. This manuscript details a comparison of multiple isothermal chemistries, assay formats and sample preparation procedures for detection of FMDV.

The analytical sensitivities of isothermal assays were equivalent to previously published results when using an FMDV RNA standard, with all RT-LAMP assay formats showing comparable analytical sensitivity to rRT-PCR (Waters et al., 2014, Howson et al., 2015) and rRT-RPA-exoRT displaying a one log_10_ reduction in sensitivity (Abd El Wahed et al., 2013). However in this study, the template of choice impacted upon assay performance with more variation evident between assays when assessing relative diagnostic sensitivity using RNA extracted from clinical samples. This is likely due to the absence of host-derived nucleic acid and/or absence of nucleic acid structures present in a RNA standards, however further work is required to confirm this. These results should be considered by researchers when evaluating new assays, to prevent the false overestimation of sensitivity and performance if this is determined using a RNA standard only.

Consistent with Yamazaki et al. (2013) who evaluated Shao et al. (2010) primers and probes (TMSC primers are based on these), rRT-LAMP-T-wet displayed the highest sensitivity of all isothermal assays, however at present this assay has only been evaluated in a wet format suitable for laboratory settings. As previously published by Howson et al. (2015), lyophilisation of rRT-LAMP-D and RT-LAMP-LFD-D reagents did not impact on the assay sensitivity or specificity; similar work needs to be carried out for rRT-LAMP-T-wet to transition this assay into a format suitable for field use. For rRT-RPA, the TwistAmp^®^ exo RT kit displayed better performance than the TwistAmp^®^ nfo kit, likely due to the addition of Exonuclease III, which contains 3ʹ-5ʹ exonuclease activity, enhancing fluorescent probe degradation during amplification. However, rRT-RPA assays consistently had reduced sensitivity compared to all RT-LAMP formats; previous studies have shown that the additional RT enzyme (RT ‘Transcriptor’, Roche) to the TwistAmp^®^ exo RT kit helped to increase assay sensitivity (Abd El Wahed et al., 2013), however this requires maintenance of the cold chain and is consequently less suitable for field-based assays.

Sample preparation remains the bottleneck of *in situ* nucleic acid detection, with current procedures being complex, time-consuming and requiring both dedicated laboratory spaces and specialised equipment (Dineva et al., 2007). This manuscript also presents a comparison of different simple sample preparation techniques across two isothermal assays. For both rRT-LAMP-d-wet and rRT-RPA-exoRT, both manual nucleic extraction kits gave comparable analytical sensitivity to automated extraction across all sample types. However, the requirement for multiple stages limits the use of these to laboratory settings, with more direct procedures required for field-based detection of FMDV.

rRT-RPA-exoRT displayed poor tolerance to inhibitors, with both sera and OP fluid inhibiting assays when added in the absence of RNA extraction. For epithelial suspensions, amplification in the rRT-RPA-exoRT assay was evident following dilution of the samples, however syringe filters were required to prevent non-specific amplification. Previous studies have indicated that LAMP shows increased tolerance to inhibitors compared to PCR (Poon et al., 2006, Waters et al., 2014). In this study, rRT-LAMP was performed in the absence of RNA extraction for multiple sample types (epithelial suspensions, sera and OP fluid). However, the analytical sensitivity of rRT-LAMP reactions was reduced when using simple sample preparation methods compared to RNA extraction. For instance, simple dilution of epithelial suspensions in NFW (1 in 5, 1 in 10 or 1 in 20) resulted in an one log_10_ decrease in sensitivity, consistent with the dilution factor. For serum, the use of Chelex^®^ 100 (plus a heat stage) produced the best results for direct rRT-LAMP-d-wet, with a one log_10_ decrease in sensitivity compared to extraction methods (again consistent with dilution factor and previous publications (Howson et al., 2015)). Chelex^®^ 100 has previously been reported as an extraction method for serum, as the ion-exchange resin removes the effect of inhibitors (Walsh et al., 1991, Ochert et al., 1994); the same effect was not seen for the other sample types. Direct sample preparation methods for OP fluid resulted in a two log_10_ decrease in the rRT-LAMP-d-wet assay LOD compared to extraction methods, suggesting partial assay inhibition in addition to the effects of dilution factor, consistent with previous publications (Howson et al., 2015).

Although the evaluation of assay performance is specific to the conditions/primers tested in this analysis, the compatibility of RT-LAMP chemistry with numerous sample types offers advantages over current point of care tests by being able to detect FMDV across a larger diagnostic window of detection, in addition to displaying increased analytical sensitivity. For example, Ag-LFDs only offer equivalent diagnostic sensitivity to laboratory-based Ag-ELISAs (Ferris et al., 2009), which limits their application to the acute clinical phase of disease and samples which contain high amounts of intact virus particles (epithelium and vesicular fluid only).

## 5 Conclusions

In conclusion, this manuscript presents the first side-by-side analysis of multiple isothermal assay formats in order to define their current capabilities benchmarked against rRT-PCR. RT-LAMP assays are comparable to the diagnostic rRT-PCR when considering a cut-off value and using extraction methods. Significantly, for RT-LAMP there was only a one log_10_ reduction in sensitivity when RNA extraction was negated for epithelial tissue suspensions (diluted 1 in 5) and serum samples (treated with Chelex^®^ 100 [heat]). RT-RPA was not as sensitive as rRT-PCR (using RNA extraction), and when used in the absence of extraction techniques was prone to inhibition across OP fluid and serum samples. With standard extraction techniques not a viable option for pen-side testing, the ability to perform RT-LAMP in the absence of RNA extraction over a large diagnostic detection window, provides a realistic means to rapidly confirm positive FMD cases close to the point of sampling.

## Conflict of interest

Masayoshi Takahashi and Hiroaki Goto are employees of Toshiba Medical Systems Corporation. All laboratory work and evaluation was undertaken at The Pirbright Institute (TPI), and no financial support was provided from Toshiba Medical Systems Corporation to conduct this study.
